# Missed opportunities for timely diagnosis of pediatric lupus in South Africa: a qualitative study

**DOI:** 10.1186/s12969-017-0144-6

**Published:** 2017-02-23

**Authors:** Laura B. Lewandowski, Melissa H. Watt, Laura E. Schanberg, Nathan M. Thielman, Christiaan Scott

**Affiliations:** 1 National Institute of Arthritis, Musculoskeletal, and Skin Diseases, NIH, DHHS, 9000 Rockville Pike, Building 10, 12 N248 Room 28, Bethesda, MD 20892-1102 USA; 2 Duke Global Health Institute, Duke University, 310 Trent Drive, Durham, NC 27710 USA; 3 Pediatric Rheumatology, Duke University Medical Center, 2301 Erwin Road, Durham, NC USA; 4 Red Cross War Memorial Children’s Hospital, University of Cape Town, Klipfontein Road, Rondebosch, Cape Town, Western Cape South Africa

**Keywords:** Lupus, Pediatric SLE, Africa, Access to care, Chronic illness, Qualitative

## Abstract

**Background:**

Systemic Lupus Erythematosus (SLE) is a serious multisystem autoimmune disease, which is more aggressive in children and people of African descent. In South Africa, pediatric SLE (pSLE) patients are at high risk for severe disease. Similar to pSLE worldwide, South African children and adolescents with SLE require subspecialized medical care. The aim of this study is to describe the care-seeking experiences of families and examine factors that contribute to delays in the diagnosis of pSLE. Specifically, we sought to identify factors to inform interventions that support the timely referral and diagnosis of pediatric SLE patients in South Africa.

**Methods:**

In-depth, semi-structured interviews were conducted with 22 caregivers of pSLE patients recruited from two government hospitals in Cape Town, South Africa in 2014. Interviews were audio-recorded, transcribed, and analyzed for themes related to barriers to diagnosis.

**Results:**

Six themes were identified and classified as either caregiver or health system barriers to diagnosis. Caregiver barriers included lack of knowledge regarding SLE, financial difficulties, and the social stigma of SLE. Health system barriers were lack of trained staff, a complex medical system, and misdiagnosis.

**Conclusion:**

Caregivers reported missed opportunities for diagnosing pSLE in their children. Raising public awareness may improve caregiver awareness and reduce stigma of pSLE. Improving family education at diagnosis holds potential to increase patient-physician trust and mitigate fear. Education modules for primary care providers at initial point of contact with the health care system may improve recognition of early pSLE and facilitate expedited referral to a specialist.

**Electronic supplementary material:**

The online version of this article (doi:10.1186/s12969-017-0144-6) contains supplementary material, which is available to authorized users.

## Background

Systemic Lupus Erythematosus (SLE) is an autoimmune disease characterized by multisystem episodic inflammation leading to severe morbidity and mortality. Prior to the discovery of corticosteroids, SLE was universally fatal; life expectancy today is still 10–30 years less than for the general population [1]. In high-income nations, patients of African descent have increased rates of lupus nephritis and end stage renal disease (ESRD), with mortality rates two to three times greater in African Americans than Caucasians [2]. Both genetic risk and social determinants of health influence these outcomes [3, 4]. Individuals diagnosed with SLE during childhood accumulate more irreversible damage than adult counterparts [5, 6]. Therefore, children in Africa may be at risk for severe disease, but little is known about this population [7–9]. The few studies of SLE in low-income nations have consistently documented worse disease outcomes compared with high-income nations [9–11]. Adult SLE studies in South Africa demonstrate a 5-year survival rate of 67-85%, compared with 95% in the United States [12, 13]. A recent study of pediatric SLE (pSLE) patients in South Africa described high rates of lupus nephritis and increased risk for progression to ESRD, dialysis and transplant [9], but did not explore the role of diagnostic delay on outcome.

South Africa is a low- to middle-income nation and health-care leader in Africa, yet health care disparities exist due to racial, political and economic inequities that have persisted after apartheid [14]. The burden of infectious diseases, trauma, and the HIV epidemic compromises access to health care for patients with chronic illness [15]. South Africa is highly underserved in pediatric rheumatology with only five registered pediatric rheumatologists in a country of over 18 million children [16]. Based on estimates from the USA, South Africa would require 40 additional pediatric rheumatologists to provide adequate care for its patient population [17].

Timely diagnosis of pSLE significantly improves long-term outcomes in children. Early diagnosis and treatment reduces organ damage, prevents renal failure, and improves survival [18, 19]. Previous studies in South Africa have demonstrated barriers to health care access, from poor road infrastructure to an inadequate medical referral system [20, 21]. U.S. studies of adult SLE patients highlight socioeconomic status, education, and geographic location as determinants of access to care, showing such disparities influence long term outcomes [22, 23].

An in-depth understanding of the path to pSLE diagnosis in South Africa is necessary to identify barriers to timely diagnosis and referral for specialized care, but has not been addressed in the available literature. Our study used qualitative interviews to contextually explore the diagnosis experience for families and identify gaps in health care access for children with pSLE in South Africa.

## Methods

### Setting

Participants were recruited through pediatric and adult rheumatology clinics in two government-funded tertiary hospitals and one private practice clinic in Cape Town, South Africa. A single pediatric rheumatology provider (CS) provides care at both hospitals, and a general rheumatologist provides care at the private clinic.

### Subject recruitment

Caregivers of patients diagnosed with pSLE (fulfilling >4 of 11 ACR SLE classification criteria [24] prior to age 19 years) were eligible to participate. A list of eligible caregivers was generated from an established registry of patients with pSLE at two tertiary care, government-funded university hospitals (Red Cross War Memorial Children’s Hospital [*n* = 18]; Groote Schuur Hospital [*n* = 10] and one private medical center (Winelands Rheumatology Centre [*n* = 2]) in Cape Town, SA.

Of 72 total SLE patient caregivers, 30 were successfully contacted. Study personnel approached caregivers in-person, by phone, or by email. Of the 30 caregivers which were contacted successfully, 26 accepted, 4 declined, 4 were unable to schedule the interview due to illness or a move, and 22 were interviewed.

### Ethical review

The study was approved by the Duke Institutional Review Board (#Pro00045133) and University of Cape Town Human Research Ethics Committee (#424/2013). Written informed consent was obtained in the participant’s preferred language prior to the interview.

### Procedures

Individual in-depth interviews were performed using a semi-structured interview guide with open-ended questions exploring care-seeking behavior during the patient’s initial illness, experiences that led to a pSLE diagnosis, and specific barriers to diagnosis (Additional file 1). The interview guide was developed based on a literature review on health care access in South Africa, SLE care access worldwide, and discussion among authors [25–27]. A semi-structured interview allowed individual flexibility and targeted probing of the participant’s experience and perspective. Participants were offered the option to be interviewed at clinic (*n =* 19) or their home (*n* = 3), to reduce the burden of missed work and travel. Interviews were conducted by a female researcher (LBL) and lasted 30 to 90 min. For non-English speaking participants, interviews were conducted in their primary language (Afrikaans or isiXhosa, the only non-English languages spoken by caregivers in this study [Table 1]). Bilingual or multilingual participants were encouraged to use the language they spoke most comfortably. In cases where the language of the interview was not English (*n* = 6), a translator who was linguistically and culturally competent provided simultaneous translation.

**Table 1:** Demographic information of pediatric SLE sample (*n* = 22)

Characteristics	Number	Percent
Caregiver relation to patient		
Mother	18	81
Father	2	9
Grandmother	1	5
Foster mother	1	5
Race of caregiver		
Coloured	16	73
Black	3	13
White	2	9
Indian	1	5
Language of interview		
English	16	72
Afrikaans	3	14
Xhosa	3	14
Age of caregiver, years	Mean: 44.2	
	Range: 32–74	
Caregiver highest education level		
Less than Grade 12	8	36
Grade 12	8	36
University or higher	3	14
Not reported	3	14
Access to private transportation		
Yes	7	32
No	14	59
Unknown	2	9
Average household income per month	Mean R4,693 ($363 USD)	
	Median R1562 (112 USD)	
	Entire Range R950–R10,000 ($73–800 USD)	
Age of child SLE patient at diagnosis, years	Mean: 10.9	
	Range 7–15	
Time to diagnosis	Mean: 23.6 weeks	
	Median 7.5 weeks	
	Range 0.5–208 weeks	

Self-described participant race was reported using racial groups employed in South African population surveys: Coloured, Black, White, and Indian/Asian [16]. In South Africa, the term “Coloured” refers to a historically racially heterogeneous ethnic group possessing ancestry from various Khoisan and Bantu tribes of Southern Africa, Malaysia, Europe, and Asia; “Black” refers to those with Southern African Bantu tribal ancestry; “White” refers to those of European descent; and “Indian/Asian” refers to those of Indian or Asian ancestry.

### Analysis

Interviews were audio recorded and transcribed verbatim. The interviews were analyzed using a conventional content analytic approach to identify and organize recurrent themes related to the study aim of identifying barriers to pSLE diagnosis [28]. Analysis was conducted in five steps. First, interviews were reviewed using inductive analysis to identify emerging themes [29]. Second, major themes identified were reviewed by two authors (LBL, MHW) in order to reach consensus [30]. Third, analytic memos were written to summarize content and organize participant responses [31]. Relevant quotes were incorporated into the memos to represent family experiences obtaining a pSLE diagnosis. Fourth, memos were manually coded for primary thematic categories related to diagnostic barriers at the level of the health system or patient. For each of the themes, representative quotations were identified as examples, and corresponding memos and transcripts were revisited to contextualize participants’ words within their overall narratives.

## Results

The sample consisted of 22 participants, all primary caregivers of pSLE patients at study enrollment and included 18 mothers, 2 fathers, 1 grandmother, and 1 foster mother. The caregiver race matched the child in all cases: 16 Coloured, 3 Black, 2 White, and 1 Indian (Table 1). The monthly household income spanned a wide range (R950 to R10,000 [$70 to $730 USD], median income R1562 [112 USD]). Thirty-six percent of South Africans live below the established poverty line (R620/$43 USD per month) [32, 33]. None of the caregivers in this study reported household incomes below that level. However, the majority of caregivers (59%) were government social welfare grant recipients and would likely be below the poverty line without social grants. Eighteen percent of caregivers and patients lived in a home without running water or electricity. Of the two White caregivers, neither reported receiving a social grant. All caregivers without running water and electricity were Black or Coloured.

For the majority of participants, there was a significant delay between onset of symptoms and a diagnosis of pSLE. The mean time to diagnosis was 23.5 weeks (median 7.5 weeks, range 0.5 to 208 weeks). At the time of diagnosis, pSLE patients had seen multiple providers, with increasing symptom severity. The majority of pSLE patients in this study (63%) had severe symptoms such as acute renal failure, stroke, or pericarditis requiring intervention at the time of diagnosis. Barriers to timely diagnosis occurred at the level of the caregiver and the health system. Below, we present themes within each level.

### Barriers at the caregiver level

We identified caregiver barriers, including lack of knowledge regarding SLE, financial obstacles to medical care, and the social stigma of a SLE diagnosis. Representative quotes are presented in Table 2.

**Table 2:** Caregiver level barriers: themes and illustrative quotations

Theme: Caregiver Knowledge Gap as a Patient Level Barrier	
Subject 5	“He had chest pain and headache. We thought he fell on the stove, nobody saw him fall on the stove. They assumed it was because he was in pain and couldn’t move. It was 3 to 4 days he had this pain and couldn’t get out of bed.”
Subject 18	“She had swollen feet. She had been crossing through dirty water. I thought her feet became swollen from playing in that water.”
Subject 19	“At first I thought she was just plain lazy, because whenever I told her to do the dishes she would sit…she was always sleepy…she was tired, and she was itching, her hands were like blotches on the skin.”
Theme: Financial Barriers for Caregivers	
Subject 2	“I did get a [hospital] bill. I told them I don’t pay this. They said just bring proof…It’s a terrible story. You have to go for affidavits to say you’re not working and stuff like that and commissions of oath you have to get signed and then you have to take the papers in and it takes a month or two.”
Subject 10	“[Time spent at the hospital] is un-paid time- that type of thing where you have to take off.”
Subject 12	“When L- (patient) was diagnosed, I was off work for a month and when I came back they arranged a tea to raise funds for us.”
Subject 14	“The blood work, doctor and medication…it was more than 2,000 Rand ($120 USD)…I’m still paying it off.”
Subject 15	“There has been difficulty because we were not prepared for it all. Many times we had to take our bread money to go to the doctor.”
Theme: Social Stigma of sick child	
Subject 2	“There’s not a lot of [SLE] awareness…There’s more awareness of crime and anything else.
Subject 3	“I tell them that… it’s only in his joints and that it affects his joints and it’s not contagious for somebody else to get.”
Subject 7	“It’s not that she must stay away from the others…No, no, no. No she’s not sick. She’s normal.”
Subject 10	“People don’t understand. They have all sorts of ideas of what lupus is.”

### Caregiver knowledge gap in SLE

Most caregivers had no prior knowledge of SLE, and thus were unable to recognize symptoms. Caregivers recognized that their child was ill, but often mistakenly attributed the symptoms to other causes. As symptoms evolved or progressed, caregivers were motivated to seek evaluation or re-evaluation. One caregiver described a typical escalation in symptoms over time: “She went to school and she couldn’t walk… [then] she was bed ridden and she couldn’t walk at all…I tried to bathe her and she fainted, and then I knew there was big trouble.”

### Financial barriers to care

A major barrier was the financial burden of accessing health care. Caregivers spoke about the lasting financial impact of hospitalization early in the disease course. One caregiver highlighted the repercussions of paying hospital bills on household finances: “Where am I going to get the money to pay this [bill]? You understand, what am I going to eat with?” Many missed one or more days of work, for which they were not paid. In addition, caregivers had to pay for transport to and from the medical facility, as the majority of participants lacked access to private transportation (Table 1). All of the White and Indian caregivers in this study had access to private transport, while only twenty-two percent of Black or Coloured caregivers did.

### Social stigma of SLE

Many families reported that they feared social stigma due to their children’s symptoms and subsequent diagnosis of pSLE. Caregivers felt the need to hide the diagnosis from the community for fear of being stigmatized. Caregivers reported that others would think their child was contagious: “I think they steer clear because they think they can get the illness. Then I tell them that it is not [contagious] and they don’t understand.” Caregivers explained that there was no familiarity with SLE within the community; therefore, there was a lack of understanding that pSLE was a chronic, non-communicable disease.

### Barriers to care at the health system level

In addition to patient and caregiver barriers, there were also health system obstacles to obtaining a diagnosis, including the lack of trained personnel at initial care contact, a complex medical system, and misdiagnosis. Representative quotes are presented in Table 3.

**Table 3:** Health system level barriers: themes and illustrative quotations

Theme: Lack of Trained Staff	
Subject 3	“At the day hospital the one nurse…it was like she had some type of attitude with me…when I told her is there any reason you guys can give me why the nose is continuously bleeding. Then she told me to just go and sit in the waiting room…I went to doctor after doctor and nobody could give me an explanation. They didn’t take any bloods, nothing. It’s my child, I know. If I know that there is something wrong with him and for them to ask me how do I know…How can they tell me that when I’ve noticed the changes as a parent? So that’s the system down there.”
Subject 7	“[After my daughter had a stroke] they discharged us, then we always go each and every week. They took the test. They didn’t say anything [in regards to diagnosis]. They just want to check is she fine. They didn’t even give her any pills or nothing.”
Subject 12	“[At the day hospital] they asked me to come back because there was not a doctor there…They didn’t actually care about the patient. I took her to the day hospital and the nurse…asked if she was pregnant and sent her out, she did not examine her.”
Theme: Complex Medical System	
Subject 7	“I went the first time and they [nursing staff] said I wouldn’t be called [to be seen.] I went back a few days later and asked to see the social worker.”
Subject 10	“She had pain in her joints and an earache. They only looked at the earache at that point. So they said it was an infection…two or three days later she couldn’t get out of bed. I didn’t feel like that [respected] because I complained about both things, the joint pain and the earache, and it was sort of like what do you want me to look at first sort of thing. If you take a child to a doctor you like them to see everything.”
Subject 16	“When we went to see the doctor there and at the time I was pregnant with my other daughter, it was very irritating. I took her to the doctor, they took blood and everything and then they told me I must come to [Children’s hospital] for an appointment.”
Subject 18	“That is when the doctor said the child is sick and must go to another hospital. They did all the tests. Then they sent her home. They told me whatever comes up bring the child back here…I went to the other GP…to mention about the child having a clotting problem…the GP told me ‘No, we are not going to do anything.”
Theme: Misdiagnosis	
Subject 1	“The GP said to me…it’s an insect bite and she gave me something, not even an antibiotic…then his other foot started swelling. At that stage he couldn’t wear his shoes anymore…I had to take him in and out of the bus because he couldn’t go upstairs. I went to the clinic…she said immediately take the letter up to [Children’s hospital] and she said he must stay at the hospital.”
Subject 2	“She had a platelet count of 7 and they admitted [her to the] oncology [unit.] One day they tell you one thing, then the next day it’s something else…Every day it was something different for quite some time. It was frustrating.”
Subject 15	“They put us in an emergency ambulance, because her [hemoglobin] was very low. They saw a specialist in [hometown]. They thought she had Leukemia, a rare cancer of the bone marrow, and then they discovered it wasn’t that. They realized that they had to find another doctor because they were worried that she was going to die so they went to [regional center]. So from there they were referred to see a doctor in Cape Town. She sent us to [the children’s hospital]… and she started getting better.”

### Lack of trained staff

The majority of caregivers went to a local primary care clinic as their first care contact, where they were seen by staff nurses or general practitioners. Poor communication of disease assessment from health care providers to caregivers was reported frequently. Many participants reported that they were discharged without further testing or treatment. Others were told to return on a day when a doctor would be present: “There was some blood in her pee. I took her to the doctor; it was on a Saturday…the doctor…gave me some medicine to give her and said if nothing changed I should come back on Wednesday. We went back…they kept her for a week but they couldn’t find out what was wrong …so they made an appointment at [the Children’s hospital].”

### Complex medical system

**Fig. 1:**
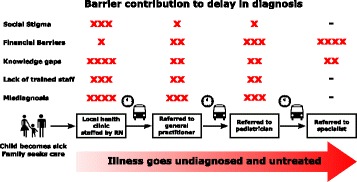
Caregiver experience of the complex referral system in South Africa

In South Africa, formal referral is required to access subspecialty care, including pediatrics (Fig. 1). Most caregivers first went to a local clinic or day hospital for care, returning several times to obtain a referral to a pediatrician: “[We took him for care] …quite a few times. I explained to [the doctor in the emergency room] the condition and I showed her everything and she told me I must come on a Tuesday. The pediatricians will be there.” Often, they reported that they were referred without explanation of symptoms or possible diagnosis.

In two rare cases, caregivers reported that pSLE was recognized immediately. One patient was already in rheumatology care for autoimmune disease and was diagnosed and managed efficiently. Another patient (the child of two physicians) went directly to a private physician and was diagnosed within days. These outlying cases provide a stark contrast to the difficulty most participants had navigating the complex medical system.

### Misdiagnosis

The majority of caregivers (16/22) reported misdiagnosis at least once and frequently encountered a series of misdiagnoses, even after hospitalization. In some cases, the misdiagnosis was frightening and grave (e.g. cancer); in others, it was less significant (e.g. insect bite or allergies) and the child was sent home without treatment. Following misdiagnosis, children suffered on-going symptoms resulting in a return to medical care. One caregiver described relocating across the country to obtain a diagnosis: “She woke up with blood in her mouth. I took her to the clinic and they said she has an allergy… She had blood on her nose and started vomiting blood and then we took her three different places. And then she had the stroke…it was on this [left] side. …They said maybe it is cancer…they didn’t find cancer… her hair was falling out and she was starting to bleed…I traveled by bus [from the Eastern Cape to Cape Town.] It was only me, her, and my little one.”

## Discussion

This study highlights missed opportunities for a timely diagnosis in a pediatric SLE population in South Africa from the patient caregiver perspective. The difficulties this group of caregivers experienced obtaining a diagnosis suggest that there may be a population of children with SLE in South Africa who never reach a rheumatologist. Timely diagnosis of pSLE is important to prevent long-term damage and improves outcomes and life expectancy in patients with renal disease [18, 34]. SLE patients in South Africa are at high risk for renal disease and have higher mortality rates than patients in Europe and North America [9, 35, 36]. Our findings suggest the poor health outcomes may be partly explained by delays in diagnosis as reported by caregivers.

Caregivers in this study experienced many obstacles while navigating the health care system seeking a correct diagnosis for their child with pSLE (Fig. 1). Various factors led to delay in first care contact including lack of recognition of the severity of initial symptoms and the inability to attribute symptoms to SLE. The burden of travel and long clinic wait times required caregivers to miss work. All caregivers without household water or electricity were of African descent, suggesting these caregivers were more likely to face financial burdens which would be aggravated by missing income. Almost all caregivers used public transportation to access the clinic as they did not own or have access to a private vehicle. Most families used taxis, the least expensive but most dangerous form of transportation in Cape Town [37, 38]. The “hidden costs” of access go beyond clinic fees or medication costs, and may have caused added delays in seeking care [25].

Beyond the financial challenges to pSLE care access, social stigma around a pSLE diagnosis was commonly reported and may have been exacerbated by lack of caregiver knowledge of SLE. Caregivers were worried that peers would avoid his or her child if pSLE was perceived as contagious. Additionally, most caregivers felt inadequately prepared to offer an explanation to others, allowing misperceptions to perpetuate.

**Fig. 2:**
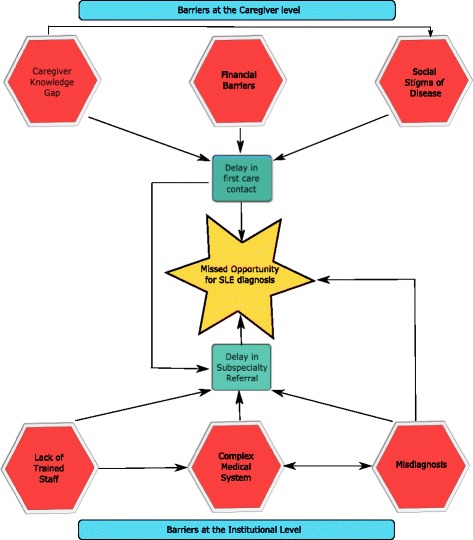
Interaction of the barriers to pediatric SLE diagnosis in South Africa

Once patients made contact with the health care system, institutional delays played a large role in delaying diagnosis. Similar delays have been reported for pSLE patients in other settings; for example, studies from the United Kingdom report multiple referrals prior to diagnosis [39]. While such delays are not unique to South Africa, the impact may be greater, due to provider scarcity, structure of the referral system, long clinic waits, and difficulty with care coordination between health providers [40]. Language barriers and lack of education about disease processes are also factors that have been identified previously as delaying SLE diagnosis [41, 42]. At each step of the referral process, patient level barriers (e.g. time and cost of transportation) are reintroduced and multiply. Barriers at both patient and health system may interact to magnify the length of delays (Figs. 1 and 2).

Seventy-three percent of families received an incorrect diagnosis in their early care-seeking experiences. Caregivers reported that providers disregarded symptoms requiring urgent medical attention. Misdiagnosis delays definitive referral and treatment, contributing to disease progression and organ damage. Patients’ and caregivers’ trust in the medical provider is a central component in care of chronic disease [43]. Initial delays in diagnosis may start a cycle of distrust between health system and patients that persist beyond diagnosis [44]. The frequency of misdiagnoses also suggests that young people with SLE may remain undiagnosed in the community.

The impact of poorly trained staff and initial misdiagnosis were compounded by the complexity of the medical system in South Africa. In nearly all caregiver interviews, first contact was with a nurse at a local clinic. A series of referrals was required before the patients were able to see a rheumatology specialist (Fig. 1). Patients were often displaying serious organ manifestations of pSLE, such as stroke, and the fear of dealing with these symptoms was escalated by confusion at time of diagnosis. Even when the diagnosing physician recognized the serious nature of the illness, the patient often had to wait several days to see a pediatrician and subsequently a subspecialist. One family relocated almost 600 miles from home to access subspecialty medical care.

This study has several limitations. First, it was conducted at two government centers and one private institution, all located in an urban location (Cape Town), causing potential selection bias in the study sample. However, 80% of the South African population uses the public sector for health care [45] and the only pSLE providers in the region are located at these centers, which increases the likelihood of representative sampling. We used a semi-structured interview process; therefore, we did not have equal depth across all topics, based on participant responses. These interviews were conducted months to years after SLE diagnosis, which could introduce recall bias. Caregivers of SLE patients may remember details of diagnosis differently than the general population would, as they have ongoing interaction with the disease, providers, and the health care system. The greatest limitation is that the caregivers interviewed had overcome barriers and reached diagnosis and linkage to specialized rheumatology care. This cohort had access to specific resources and exhibited remarkable resilience that may differ from families whose children with pSLE remained undiagnosed, or who have not successfully linked to subspecialized care for SLE treatment. While some of our caregivers received social grants to bolster household income, with grant assistance none were below the poverty line. Access to financial resources may have increased the likelihood of successful linkage to subspecialty care for caregivers in this study.

The findings of this study highlight possible interventions to improve diagnosis efficiency at both the caregiver and health system level. At the caregiver level, broadcast or online media-based public service announcements about SLE could increase community knowledge, facilitate timely diagnosis, and reduce public stigma of the disease [46, 47]. Caregivers would benefit from improved education at diagnosis, which is central to successfully managing chronic disease [48]. A standardized education packet, combined with web-based modules and videos, could fill the SLE education gap for caregivers of all literacy levels at time of diagnosis [49, 50].

At the health system level, inadequate provider knowledge and resulting misdiagnosis delayed care. South Africa has a great need for additional pediatric rheumatology providers. Continued efforts to train pediatric rheumatologists in South Africa are critical, but will require years to alleviate the current severe shortage. Training existing primary care providers to recognize the signs and symptoms of SLE would likely increase provider awareness and decrease time to referral. Tools such as the American College of Rheumatology’s Lupus Initiative [51] could be modified to meet the needs of South African providers and implemented in a pilot program. This free, web-based series of educational tools is aimed to improve SLE diagnosis and management amongst primary care providers in the US. Similar initiatives in the UK expose students, trainees, and general pediatricians to pediatric rheumatology and increase the diagnosis of rheumatic disease in childhood [52]. Short courses for direct instruction with subsidized travel for participants could complement on-line modules [53]. South African provider perspective on delays and interest in rheumatology-specific training are topics which require further research. Perhaps even more importantly, referral networks utilizing email systems or cell phone contact could facilitate earlier referral from primary care providers for this life threatening disease [54].

## Conclusion

Caregivers’ perspectives in navigating the pathway to an eventual diagnosis of pSLE in South Africa highlight profound delays in diagnosis, resulting in a lasting impact for the patient and caregiver. While many themes mirror findings from higher income countries, challenges are exacerbated by health care, social, and economic disparities unique to the South African setting. The gaps identified suggest opportunities to improve the efficiency and effectiveness of identifying patients with SLE in South Africa. Successful interventions that reduce the time to diagnosis in South Africa will provide a model for other resource limited nations [55]. Further investigation into the long-term impact of delays in care on disease damage and outcomes is necessary in the pediatric SLE population of South Africa.

## Additional file

Additional file 1: Qualitative Interview Guide for Pediatric SLE in South Africa. (DOCX 28 kb)

## Abbreviations

pSLE: Pediatric systemic lupus erythematosus
SLE: Systemic lupus erythematosus
USA: United States of America
