# Supplementary material for: Missed opportunities for timely diagnosis of pediatric lupus in South Africa: a qualitative study
Source: Pediatr Rheumatol Online J. 2017 Feb 23;15:14. doi: 10.1186/s12969-017-0144-6 (PMC5322669; doi:10.1186/s12969-017-0144-6)
Supplement: Additional file 1: — Qualitative Interview Guide for Pediatric SLE in South Africa. (DOCX 28 kb) [file 12969_2017_144_MOESM1_ESM.docx]

**Pediatric Lupus in South Africa**

**Qualitative Interview Guide**

1. **INTRODUCTION/PERSONAL INFORMATION**

To begin, I want to know a little about yourself.

1. What is your relationship with the patient? (example: biological parent, primary caregiver, legal guardian)
2. Please tell me about yourself, like where you were born and your background. [Probe: age, schooling, religion, race]
3. What is your home life like? [Probe: where she lives, who she lives with, relationship status, number/ages of children]
4. Please tell me about how you and your family support yourselves. [Probe: economic activities, government grants, whether there is enough money in the household]
5. **EXPERIENCE OF INITIAL PEDIATRIC SLE DIAGNOSIS**

I would like to hear about the experience of your child being sick and getting care.

1. Please tell me about how you first knew there was something wrong [Probe: Symptoms, what s/he thought was happening.]
2. Please tell me how you tried to help your child when s/he first became sick. [Probe: Story of the illness, experiences in seeking medical care, use of home remedies or traditional healers]
3. What was your experience getting care in clinics or hospitals for your child? [Probe: Experiences at hospitals/clinics, referrals, how the child was treated, delays in seeking care and getting referrals, treatment in the hospitals/clinics]
4. Please tell me about when your child was diagnosed with lupus. [Probe: how long ago, how soon after symptoms started, how soon after seeking care, where and by whom diagnosed, your reaction to diagnosis, whether she trusted the doctor who diagnosed the child **Confirm time from first symptoms to diagnosis**]
5. What happened after your child was diagnosed with lupus? [Probe: referral to specialized care, any fears/confusions about the new clinic]
6. What is your child’s health care like now? [Probe: all the places the child gets clinical care, how often the child has clinical appointments, how far they travel for clinical care and how much that costs]
7. What kind of experiences have you had of your child being hospitalized for lupus? [Probe: Number of times hospitalized, experience in the hospital, if other children, who takes care of them, cost of hospital care]
8. **EXPERIENCE LIVING WITH A CHILD WITH LUPUS**

I am interested in learning about your experiences living with a child who is sick with lupus.

- 1. Please tell me about how it has impacted your life to have a child with lupus.
  2. How has having a child with lupus impacted you and your family financially? [Probe: money spent on clinic/hospital fees, travel, ability to earn an income]
  3. How has having a child with lupus impacted your emotional well-being? [Probe: changes in her emotions, levels of stress, any anxiety/fear she feels]
  4. How has your child’s disease changed the way you think about your child or ways you treat your child? [Probe: specific examples of changes]
  5. How has your child’s disease changed the way other people in your family or community treat you or your child? [Probe: spouse/partner, other family members, neighbors.]
  6. *If other children*: How has having a child with lupus impacted the other children in in your family? [Probe: how s/he manages to take care of the other children especially when the sick child has appointments, any emotional impact]
  7. When you have to explain to someone what lupus is, what do you tell them? What about when they ask about the medicines to treat lupus?
  8. What do you think the future looks like for you and your child?

1. **SOCIAL SUPPORT AND STIGMA**

I’d like to hear how others treat you because you have a sick child.

1. Who in your life gives you support to help take care of your child? [Probe: relationship and type of support; any support that is lacking]
2. In general, how are parents with children who have serious illnesses treated in your community? [Probe: any good or bad treatments]
3. How have you been treated in your community because you have a child with lupus? [Probe: who in the community knows about the illness; any good or bad treatments]
4. **THOUGHTS, EMOTIONS, AND A CHILD WHO HAS SLE**

I am interested in learning about your thoughts and feelings about your child and the diagnosis of lupus

- 1. How often do you think about your child’s illness, and how does that make you feel? [Probe: emotions, thoughts, worries]
  2. In the few months after your child’s diagnosis, how did you feel emotionally? [Probe: has this changed since then? How so? Have her describe specific emotions, both positive and negative, including e.g., sadness, anxiety, fear, hope, anger]
  3. What memories from the time of your child’s illness do you still think about the most? [Probe: Feelings related to the memories, how often she thinks about them, who she talks to, what moments were most frightening]
  4. How has having a child with a serious illness changed the way you think about yourself? [Probe: thoughts about role as a parent]

1. **PEDIATRIC LUPUS TREATMENT PREFERENCES**

I would like to ask you some questions about how you would like your child to be cared for.

1. What things are you most hopeful about related to your child’s disease or medical treatment?

1. What things are you afraid of related to your child’s disease or treatment?
2. What questions do you have about your child’s disease, treatment, or doctors?
3. What additional support would you like from your doctors or nurses?
4. **CONCLUSION**

Now I have asked you all my questions.

1. Do you have anything else you would like to tell me about your experiences as a parent of a child with lupus?
